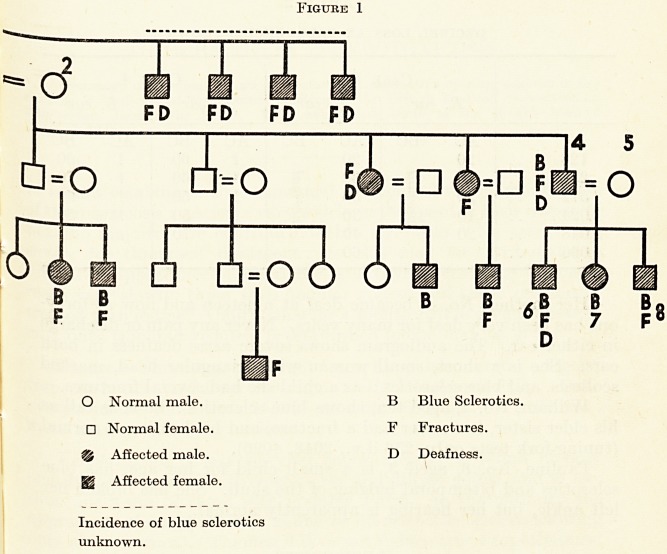# Blue Sclerotics, Fragilitas Ossium and Deafness: Report of a Family
*Four members of the family were shown at the combined Summer Meeting of the sections of Laryngology and of Otology of the Royal Society of Medicine at Bristol on July 2nd, 1948.I wish to acknowledge the help of Mr. G. F. Home, the Librarian of the Society, in the preparation of this article.—E. W.-W.


**Published:** 1948

**Authors:** E. Watson-Williams


### BLUE SCLEROTICS, FRAGILITAS OSSIUM AND DEAFNESS: REPORT OF A FAMILY*

BY

E. WATSON-WILLIAMS, *M.C.*, M.D., Ch.M.,

---

THAT a young and apparently healthy person should frequently break his bones, often from some trivial cause, seems bound to attract attention: still more, when this remarkable condition afflicts several members of one family, which is further distinguished by a startling blueness of what in others is "the white of the eye". Yet, although the condition is by no means rare, the first recognizable account appeared in 1788, when Eckman[1] described a family, three generations of which suffered from multiple fractures. Nearly a century elapsed before similar accounts were published in this country.[2]

In none of the early accounts is there any mention of the blueness of the sclerotic. Though of itself a striking phenomenon, it appears to have received no attention until 1896, when Spurway mentioned it casually at the end of an article on "Hereditary Tendency to Fracture."[3] The association of the two conditions was expounded by Eddowes in 1900.[4] Soon after this there appeared pedigrees of many families showing these two abnormalities:[5] in several of them, one or two individuals are recorded as being deaf. The importance of deafness was first shown by Bronson in 1917[6], who published two family trees: in one (Currie), among fifty-five persons, twenty suffered from fractures and had blue sclerotics, and seven of those were deaf.

In 1928 Bell[7] collected seventy-four pedigrees, with photographs, X-ray and colour drawings, illustrating the characteristic deformities. All the bones tend to be affected, the long bones especially. These

* Four members of the family were shown at the combined Summer Meeting of the sections of Laryngology and of Otology of the Royal Society of Medicine at Bristol on July 2nd, 1948.

I wish to acknowledge the help of Mr. G. F. Home, the Librarian of the Society, in the preparation of this article.—E. W.-W.

are thin, lacking density, and show typical cross-striation in X-ray films ; they are easily broken—over a hundred fractures have been recorded in one patient—and there may be little pain or swelling accompanying the fracture. Repair is normal. Bending of bones is common, with resulting deformities of limbs, spine, pelvis, etc. The patients are usually of small stature, with " triangular " skulls produced by occipital and temporal (sometimes frontal) protuberances. In addition there may be abnormal laxity of ligaments : sprains and dislocations are frequent. Sometimes the tendency to fracture appears especially in one bone or limb. Fractures may occur before or during birth : the tendency to fracture diminishes in adult life.

The blueness of the sclerotics is due to these structures being abnormally thin, or perhaps only abnormally translucent,[5,6] so that the colour of the choroid is transmitted. As a rule the eyes show no other defect, but colour-blindness[9] and conical cornea[10] have been recorded. The blue sclerotics are present in all the patients, and are transmitted as a Mendelian dominant : of those with blue sclerotics two-thirds show liability to fractures, two-thirds become deaf in early adult life and nearly half show both changes. Those with blue eyes may transmit to their offspring the tendency to fractures or to deafness, although themselves not suffering from either. The sexes are equally affected and equally transmit the defect. Apparently, however, the condition may skip a generation :[11] and isolated cases occur without discoverable affected relatives—Nager's case (below) was one such.

The deafness usually appears at about the age of twenty and resembles that of otosclerosis : Rinne's sign negative (B.C.>A.C.) and bone conduction increased. But the nerve deafness which is a late phenomenon in otosclerosis here often occurs quite early. Cleminson[7] was able to publish a section from the temporal bone of a patient under Professor Nager (Zürich). It shows bone changes of the same order as those seen in typical otosclerosis ; but whereas in the latter disease there is usually only a single focus, beginning just in front of the oval window, in Nager's case there were several foci involving not only that site, but the round window, canals, and internal meatus also. Such changes would explain the differences in deafness between this condition and " ordinary " otosclerosis.

The pathology of this condition is obscure. The blood shows no material abnormality ; the blood calcium level may be rather high.[11] The usual " explanation " is a hereditary defect in the tissues of mesoblastic origin. There is no evidence of any endocrine abnormality. No treatment is known which influences the course of the disease. These patients are not suitable for the operation of fenestration of the labyrinth.

FIGURE 1

1 1st Husband of 3.

2 2nd Husband of 3.

3 Grandmother.

4 Mother of 6, 7, 8.

5 Father of 6, 7, 8.

His parents, brother and

5 sisters normal.

6, 7, 8 Three children.

The family, four members of which I bring to your notice, is interesting because the maternal grandmother married twice (Figure 1), and her descendants by both husbands show the triad under discussion. The grandmother herself, No. 3, has white sclerotics, has not broken her bones, and is not deaf : or rather, at seventy-two, has only recently begun to be deaf. But her four sisters all became deaf early in life and all suffered from fractures. (It has not been possible to discover the incidence of blue sclerotics among these, nor among descendants of the first husband ; we may reasonably assume that at least those who suffered from fractures or deafness showed eye changes also.) By her first husband, No. 1, she had four sons and one daughter. The eldest son and his three children are affected : the elder daughter of the third son, and *her* daughter, are also affected. Information is lacking about the other members of this family.

The second husband, No. 2, was normal : by him she had three daughters and two sons. The youngest daughter is No. 4, the

FIGURE 1

○ Normal male.

□ Normal female.

Affected male.

Affected female.

B Blue Sclerotics.

F Fractures.

D Deafness.

\- - - - - - - - - - - - - - - -

Incidence of blue sclerotics unknown.

mother of the three children shown. Several of *her* nephews and nieces (on her own side of the family) and her grandniece, as well as her two brothers, are known to be affected. No. 5, the husband of No. 4, is normal, as are his brother and five sisters; I have not been able to find out anything about their descendants.

Gwyneth, No. 6, aged nineteen, came to me recently because she has begun to be deaf in the left ear during one year. She knows no cause for this, but "it runs in the family". She is a slim, dark, short girl (4 ft. 8 in.) with triangular head and bright blue sclerotics. She has broken her bones six times (left leg three times): X-rays show typical changes throughout the skeleton, but no abnormal deposit of callus. The audiogram (Table 2) shows loss of hearing in both ears, more in the left, by air conduction; the loss is greater for the high frequencies. (In typical otosclerosis the higher frequencies tend to escape, at least at first.) The bone conduction is normal. No tinnitus, membranes appear normal; never any ear-ache or discharge.

TABLE 2

DECIBEL LOSS AT FREQUENCIES SHOWN

| | Case 6 | | | | Case 4 | | | |
|---|---|---|---|---|---|---|---|---|
| | *R. ear* | | *L. ear* | | *R. ear* | | *L. ear* | |
| | AC | BC | AC | BC | AC | BC | AC | BC |
| 128 .. | 20 | | 20 | | ? | 60 | ? | 60 |
| 256 .. | 10 | | 30 | | ? | 60 | ? | 70 |
| 512 .. | 20 | *normal* | 30 | *normal* | ? | 50 | ? | 60 |
| 1,024 .. | 10 | | 30 | | ? | 50 | ? | 50 |
| 2,048 .. | 20 | | 40 | | | 70 | | |
| 4,096 .. | | | 60 | | | | | |

Her mother, No. 4, became deaf at nineteen and now at forty-one has been very deaf for many years. Never any pain or discharge in either ear. The audiogram shows severe *nerve* deafness in both ears. She is a short, small woman with triangular head, marked scoliosis, and blue sclerotics ; as a child she had several fractures.

William, No. 7, aged ten, shows blue sclerotics : he is as tall as his elder sister, has never had a fracture, and his hearing is normal (tuning-fork tests only, 256 d.v., 2048, 4096).

Pauline, No. 8, aged 3, is a small child for her age, has blue sclerotics and bitemporal bulging of the skull. She has broken her left ankle, but her hearing is apparently normal.

### REFERENCES

1 Eckman, O. J., *Descriptio . . . osteom alaciae.* Upsala, 1788.

2 Jones, J., *B.M.J.*, 1870, i, 86. Greenish, R. W., ib., 1880, i, 966. Pritchard, O., *Lancet*, 1883, ii, 394.

3 Spurway, J., *B.M.J.*, 1896, ii, 844.

4 Eddowes, A., ib., 1900, ii, 222.

5 *e.g.* Harman, B., *Ophthalmoscope*, 8, 1910, 559.

6 Bronson, E., *Edin. M.J.*, XVIII, 1917, 240. Fraser, J. S., *Proc. R. Soc. Med.*, Sect. Otol., XII, 1919, 126.

7 Cleminson, F. J., ib., XX, 1927, 471.

8 Bell, J., *Treasury of Human Inheritance*, London, 1928, Vol. II, Part 3, 269.

9 Riddell, W. B., *Annals of Eugenics*, 10, 1940, 1.

10 Behr, *Klin. Monatsbl. f. Augenh*, XVI, 1913, 281.

11 Kersley, G. D., *St. Bart. Hosp. Rep.*, LXVIII, 1935, 159.

## Figures and Tables

**Figure 1 f1:**
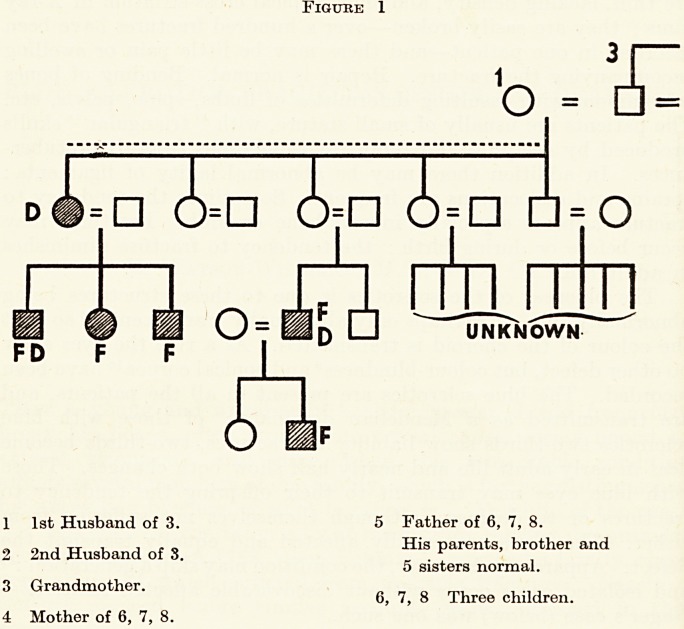


**Figure 1 f2:**